# Thermal resistance and high-performance microwave decontamination assessment of *Bacillus* endospores isolated from food-grade herbal extracts

**DOI:** 10.1371/journal.pone.0261988

**Published:** 2021-12-28

**Authors:** Armin Tarrah, Shadi Pakroo, Milena Carlot, Camilla Nesto, Antonella Cirillo, Angiolella Lombardi, Viviana Corich, Alessio Giacomini

**Affiliations:** 1 Department of Agronomy Food Natural Resources Animal and Environment (DAFNAE), University of Padova, Legnaro, PD, Italy; 2 Interdepartmental Centre for Research in Viticulture and Enology (CIRVE), University of Padova, Conegliano, TV, Italy; 3 Agripharma Società Cooperativa Agricola via Prima Strada 11, Vescovana, PD, Italy; University of Connecticut, UNITED STATES

## Abstract

Generally, endospore contamination can occur from different sources during product manufacturing in many industries and therefore lower its quality by affecting physicochemical properties and shelf-life. Bacterial endospores can germinate inside the product and produce several enzymes, which can cause several undesirable changes. This study assessed the spores thermal resistance and applied a microwave decontamination technique toward herbal extracts (*Tilia tomentosa* and *Centella asiatica*) containing ethanol or glycerol. Based on 16S rRNA analysis, the detected contaminant endospores belonged to different *Bacillus* species, namely *B*. *subtilis*, *B*. *zhangzhouensis*, and *B*. *pumilus*. The thermal resistance assessment using inoculated endospores in the actual products revealed *B*. *pumilus* T2 as the most resistant endospore to the heat treatments tested in both *T*. *tomentosa* and *C*. *asiatica* extracts. Finally, a high-performance microwave technique was used to decontaminate *T*. *tomentosa* extract against the mixture of *Bacillus* spores. Results from the microwave technique indicate that the increase of temperature from 100°C to 105°C not only decontaminated the product but also could dramatically decrease the effective thermal treatment time (10 times), which can benefit the product quality. The results provided in this study considerably contribute to improving an original decontamination method for products containing glycerol and ethanol with the most negligible effect on product quality.

## Introduction

Plants that are used for medicinal or aromatic properties are defined as medicinal and aromatic plants (MAPs) [1]. Herbal extracts from these plants have been utilized by several industries for their beneficial properties throughout history [2]. Nowadays, they are also considered by pharmaceutical and food companies, and for cosmetic purposes, so the definition of medicinal, aromatic, and cosmetic (MAC) plants can also attribute to such plants as well [1]. *Tilia tomentosa* and *Centella asiatica* are two MAC plants whose extracts have been used for such purposes [3]. Considering the wide industrial applications of herbal extracts, the need for high microbiological quality and absence of preservatives has been a big challenge since microbes normally contaminate these plants and are not easily removable, particularly considering spore-forming bacteria that are always present [4].

*Bacillus* endospores have been the most frequently reported contaminant in different industrial plants [4]. Different *Bacillus* species can undergo a unique sporulation process when faced with harsh conditions, such as adverse environment or starvation of nutrients [5]. Endospores are resistant microbial forms with remarkable characteristics that distinguish them from vegetative cells, especially the spore structure and lack of metabolism [6]. Endospores can tolerate extreme environments, such as concentrated chemicals, very low pH, and high temperatures, making their elimination problematic for many industries [4]. Typically, endospores that are abundantly present in the soil, air, and water [7] can contaminate products during manufacturing through different sources such as working personnel, raw materials, and equipment [8–10]. Such contaminations can lower the quality and seriously affect physicochemical properties as well as the shelf-life of products [4]. Besides, *Bacillus* endospores can germinate and secrete numerous enzymes [11]. They are very well-known for their ability to produce amylase, protease, lipase, and other enzymes, which can cause many undesirable changes in the final products that represent a substantial problem for food, pharmaceutical, and cosmetic products [12–15].

Unfortunately, getting rid of contaminating spores is challenging, especially as the high temperatures needed for spore killing can negatively affect product quality. Therefore, optimizing a proper decontamination method with the lowest effect on product quality could be crucial for many industries. Moreover, it must be noticed that consumers dislike the presence of chemical additives, and therefore, an optimized physical treatment could both preserve product quality and satisfy consumers’ expectations. The microwave technique has been used in different industries for microbial inactivation [16, 17]. This approach can generate heat and drive it more uniformly inside the cells through electromagnetic waves, resulting in cell death in a much shorter time with respect to traditional heat treatments [18]. The present study aimed to assess the thermal resistance of *Bacillus* endospores isolated from aqueous or alcoholic plant extracts and to investigate the potentiality of high-performance microwave techniques to inactivate them.

## Materials and methods

### Microbial analysis and isolation of *Bacillus* endospores

Sealed bottles containing *Tilia tomentosa* and *Centella asiatica* extracts were provided by Agripharma company. *Tilia tomentosa* extract was obtained by extraction from dry leaves using a 1:1 water: glycerin solution, while *Centella asiatica* extract was obtained by extraction of leaves with ethanol solution and concentration under vacuum at room temperature. Microbiological analysis of herbal pharmaceutical extracts (*Tilia tomentosa* and *Centella asiatica*) was performed by plate count analyses for the following microbial categories: total mesophilic microorganisms were counted on PCA (Plate Count Agar, Difco, MD, USA) medium, incubated at 30°C for 72 h; *Enterobacteriaceae* were enumerated on VRBG agar (Violet Red Bile Glucose Agar, Difco, MD, USA) after incubation at 37°C for 24 h; yeasts and molds were counted on DRBC (Dichloran-Rose Bengal Chloramphenicol Agar, Difco, MD, USA) incubated at 25°C for 72 h. Ten milliliters from each herbal extract were homogenized with 90 mL of sterile phosphate-buffered saline (PBS; NaCl 8.0 g/L, KCl 0.2 g/L, Na_2_HPO_4_ 1.44 g/L, KH_2_PO_4_ 0.24 g/L, pH 7.4), and serial dilutions were plated on solid media using the relative International Standard Organization (ISO) procedures [19].

### Amplification and sequencing of 16S rRNA

Genomic DNA from selected bacterial colonies was extracted using the DNeasy PowerSoil Microbial Kit (Qiagen, Valencia, CA, USA) according to the manufacturer’s instructions. DNA quality and quantity were assessed using a Spark 10M spectrophotometer (Tecan Trading AG, Männedorf, Switzerland). Microbial identification was obtained through 16S rRNA sequencing using the universal primers 27F (5′ GAGTTTGATCNTGGCTCAG 3′) and 519R (5′ GWNTTACNGCGGCKGCTG 3′) [20, 21] (BMR Genomics, Padova, Italy). Sequencing of amplified products was done as previously described by Clarridge and Han [21, 22]. Finally, the high-quality 16S rRNA sequences were searched against those available in the GenBank using the BLASTN program at the National Center for Biotechnology Information (NCBI) server [23].

### Bacterial sporulation and quantification

*B*. *subtilis* C5, *B*. *zhangzhouensis* M1, and *B*. *pumilus* T2 were grown on 2×SG agar medium (16 g/L Difco Nutrient broth, 2 g/L KCl, 0.5 g/L MgSO_4_, 17 g/L agar, 1 mL Ca(NO_3_)_2_ (1 M), 1 mL MnCl_2_·H_2_O (0.1 M), 1 mL FeSO_4_ (1 mM), 2 mL glucose 50% (w/v), pH: 7.0) [24] and left for 10 days at 35°C to allow endospore production.

After incubation, under sterile conditions, the endospores were harvested from the surface of the 2×SG agar medium using 10 ml of cold distilled water and sterilized swabs. Then, endospores suspensions were centrifuged at 5500 g for 10 min at 4°C, and the pellet was washed 3 times with cold sterile PBS. Finally, based on International Standard Organization (ISO) protocols [19], the collected pellets were used for endospore enumeration in three different ways; using a heat treatment (at 80°C for 10 min), without heat treatment, and by direct enumeration under the microscope to determine the quantity and purity of produced spores.

### Thermal resistance determination

Each herbal product was inoculated separately with approximately 10^4^ endospore (per mL) of *B*. *subtilis* C5, *B*. *zhangzhouensis* M1, and *B*. *pumilus* T2. A sample containing a mixture of all three *Bacillus* endospores (10^4^ of all endospores per mL) was included to mimic actual product contamination conditions. Then, periods of heating at the temperatures of 90°C for 10 min and 100°C for 1, 2, 5, and 10 min were applied by using a thermocycler (Bio-Rad, Hercules, California, United States), followed by rapid cooling and plating on Plate Count Agar (PCA). The experiment was performed using three technical and two biological replicates.

### High-performance microwave treatment

**Fig 1:**
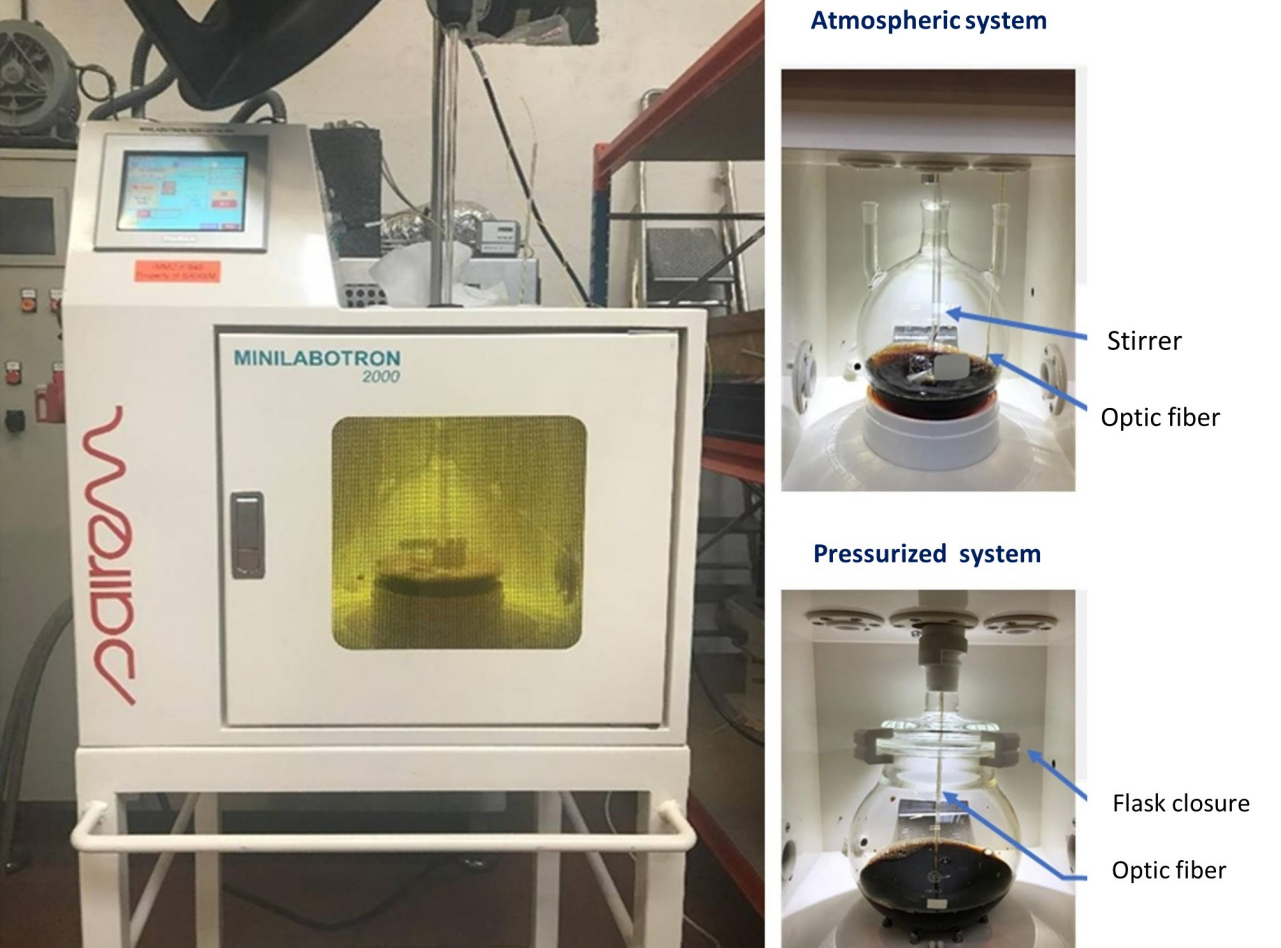
High-performance microwave-assisted equipment Minilabotron 2000 used in this study.

**Fig 2:**
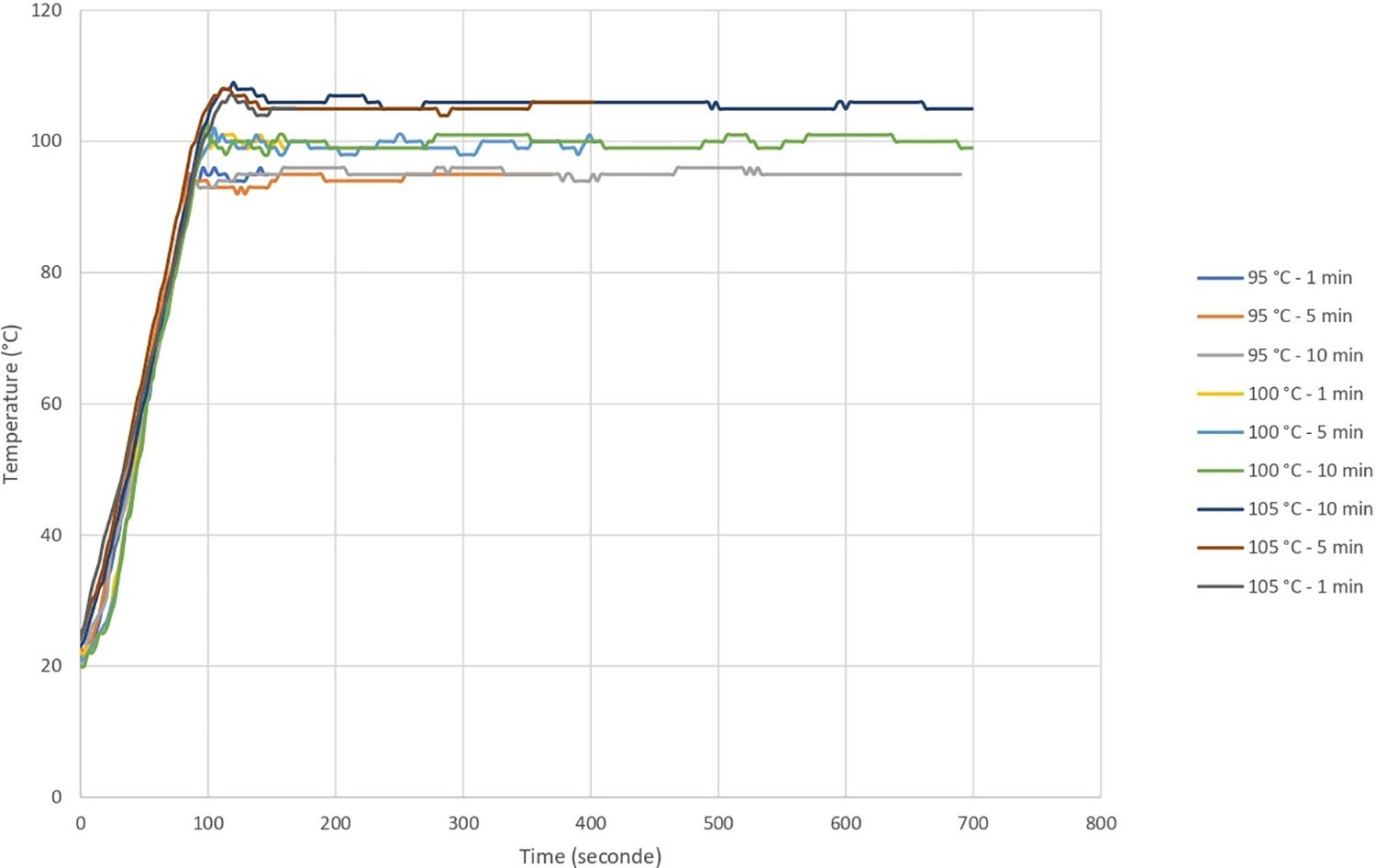
Recorded temperatures during sample thermal treatments.

High-performance microwave-assisted equipment Minilabotron 2000 (SAIREM, Décines-Charpieu, France) with 2-kW output power and an operating radio frequency heating at 2450 MHz was used for treating the *Tilia tomentosa* extract, which was inoculated with approximately 10^4^ endospores of *B*. *subtilis* C5, *B*. *zhangzhouensis* M1, and *B*. *pumilus* T2 (10^4^ endospore mixture). We used 500 mL *T*. *tomentosa* extract for the high-performance microwave treatment at various temperatures (95°C to 105°C) and periods (1 to 10 min), followed by cooling and plating as explained above. For the temperatures 95°C and 100°C, an open chemical flask was used (atmospheric pressure system), while for the temperature of 105°C, we used a closed chemical flask to create a pressurized system (Fig 1). The temperature was recorded using IR-temperature measurement inside the oven and thermocouple via the fiber optic present inside the flask (Fig 2). All experiments were performed in triplicate. Data were analyzed using a two-way analysis of variance (ANOVA). Tukey’s test was used as a *post hoc* analysis as well.

### Statistical analysis

Data were analyzed using a two-way analysis of variance (ANOVA). Tukey’s test was used as a *post hoc* analysis by the GraphPad Prism software (version 7, GraphPad Software, Inc., San Diego, CA, United States).

## Results and discussion

*C*. *asiatica* is an herb that is widely applied to treat a variety of diseases in the world due to the possession of triterpenes such as madecassoside, asiatic acid, asiaticoside, and madecassic acid [25]. *C*. *asiatica* is grown in many different places such as Sri Lanka, Madagascar, Pakistan, India, South Africa, Eastern Europe, and its extract has been used to treat inflammation and to speed wound healing [25, 26]. *Tilia tomentosa* (lime tree) is another type of medicinal herb, a tree native throughout Europe, and is widely used as a sedative, which promotes relaxation and relieves anxiety symptoms due to the presence of different flavonoids compounds [27]. In preparing these two herbs, two different approaches are used: *C*. *asiatica* extract is made by using ethanol as co-solvent, whilst the *Tilia tomentosa* extract is prepared by applying the glycerol co-solvent due to the different compounds in this herb [28], which make it more appropriate for children and alcohol sensitive people.

The results of total mesophilic counts revealed a microbial load of 10^4^ CFU/mL for both herbal extracts, whereas the analyses of *Enterobacteriaceae* and yeasts/molds gave values of viable microorganisms below the detection limit (<1 CFU/mL). On PCA plates, several different colony morphologies were present. One colony of the three most represented types of colonies was randomly picked, purified, and subjected to Gram staining, catalase, and oxidase tests to obtain a preliminary characterization. The first type was opaque and off-white, the second was rough, opaque, slightly yellow, and the last was cream-white and circular. All isolates were Gram-positive, rod-shaped, catalase-positive, and oxidase-negative, with characteristics compatible with the *Bacillus* genus, a widespread soil inhabitant. Based on 16S analysis, the three isolates were assigned to different species, namely, *B*. *subtilis*, *B*. *zhangzhouensis*, and *B*. *pumilus*. (Table 1). It has to be considered that, during the preparation of herbal extracts, microbiological contaminations naturally occur from plants that had already been contaminated with endospores [29, 30]. Secondary contamination can also happen due to poor storage conditions [31].

**Table 1:** Partial 16S rRNA sequencing of *Bacillus* strains and strain classification.

Strain ID	E-value	Identity (%)	Species (16S rRNA gene analysis)	Accession number
C5	0.0	99.22	*Bacillus subtilis*	MN865860
M1	0.0	99.80	*Bacillus zhangzhouensis*	MN865862
T2	0.0	97.48	*Bacillus pumilus*	MN865861

The results of sporulation and endospore enumeration with and without the heat treatment at 80°C for 10 min, did not indicate any differences, confirming that no viable vegetative cells remained after the incubation. The data obtained from sporulation (*B*. *subtilis* C5: 9.10±0.071; *B*. *pumilus* T2: 9.86±0.013; *B*. *zhangzhouensis* M1: 9.82±0.014 log spores/mL) were used for sample initial target inoculation (10^4^ spores/mL).

Plant extracts were subjected to thermal treatments to assess endospores’ heat resistance. Results of spore survivability for *T*. *tomentosa* are reported in Table 2 and for *C*. *asiatica* in Table 3.

**Table 2:** Effect of different temperature/time combinations on endospores inoculated in *Tilia tomentosa* extract.

Treatments	*Bacillus* spp. endospores			
	*B*. *subtilis* C5	*B*. *pumilus* T2	*B*. *zhangzhouensis* M1	mixture of endospores
Initial number	4.14±0.05* (100%)**	3.86±0.07 (100%)	4.23±0.02 (100%)	4.18±0.12 (100%)
10 min (90°C)	3.83±0.03 (49%)	3.64±0.03 (60%)	4.06±0.04 (66%)	3.99±0.02 (64%)
10 min (100°C)	<1.00	<1.00	2.34±0.05 (1%)	<1.00
5 min (100°C)	<1.00	1.80±0.04 (1%)	2.87±0.08 (4%)	<1.00
2 min (100°C)	3.65±0.13 (32%)	3.79±0.06 (84%)	3.60±0.05 (23%)	3.93±0.03 (55%)
1 min (100°C)	4.05±0.14 (83%)	3.86±0.05 (99%)	4.01±0.01 (60%)	4.05±0.03 (73%)

* Colonies were counted as CFU/ml and results are expressed as the mean (log_10_) ± SD (n = 3) of surviving endospores. The plates without colony were rechecked as CFU/mL to detect any surviving endospores, and the values remained the same (<1.00).

**The values reported in the parentheses indicate the percentage of surviving endospores.

**Table 3:** Effect of different temperature/time combinations on endospores inoculated in *Centella asiatica* extract.

Treatments	*Bacillus* spp. endospores			
	*B*. *subtilis* C5	*B*. *pumilus* T2	*B*. *zhangzhouensis* M1	mixture of endospores
Initial number	4.14±0.05* (100%)**	3.86±0.07 (100%)	4.23±0.02 (100%)	4.18±0.12 (100%)
10 min (90°C)	3.73±0.02 (40%)	3.59±0.03 (54%)	4.02±0.03 (61%)	3.93±0.02 (56%)
10 min (100°C)	<1.00	<1.00	<1.00	<1.00
5 min (100°C)	<1.00	<1.00	<1.00	<1.00
2 min (100°C)	3.54±0.06 (25%)	3.72±0.02 (73%)	3.53±0.02 (20%)	3.85±0.02 (46%)
1 min (100°C)	4.05±0.10 (83%)	3.86±0.02 (100%)	4.01±0.04 (60%)	4.02±0.02 (69%)

* Colonies were counted as CFU/ml and results are expressed as the mean (log_10_) ± SD (n = 3) of surviving endospores. The plates without colony were rechecked as CFU/mL to detect any surviving endospores, and the values remained the same (<1.00).

**The values reported in the parentheses indicate the percentage of surviving endospores.

Regarding *Tilia tomentosa* extract (containing 30% glycerol), *B*. *zhangzhouensis* M1 revealed the highest heat resistance at 100°C for the time intervals 5 and 10 min as compared to *B*. *subtilis* C5 and *B*. *pumilus* T2 (Table 2). However, when considering the heat resistance at 100°C for 1 and 2 min, *B*. *pumilus* T2 showed the highest heat resistance among different types of endospores by 99% and 84% survival rate, respectively. However, heat treatment using 90°C for 10 min did not reduce the number of endospores significantly.

The endospores in *C*. *asiatica* extract (containing 30% ethanol) showed different behavior. In *C*. *asiatica* extract, *B*. *zhangzhouensis* M1 revealed the lowest survival at 100°C while in *T*. *tomentosa* extract, it had shown much higher resistance compared to the other endospores (Table 3), which can be linked to the simultaneous effect of ethanol and heat in this product and the sensitivity of *B*. *zhangzhouensis* M1 endospores to ethanol content. In contrast, *B*. *pumilus* T2 had the least thermal susceptibility (Table 3). It has been proved that a combination of ethanol and heat can affect the inner membrane permeability of some bacterial endospores and significantly reduce their survivability during the treatment [6]. Also, in previous work, Setlow *et al*. [32] had shown that the simultaneous use of heat and ethanol could significantly decrease the resistance of some endospores

On the other hand, we have used the high-performance microwave technique to decontaminate the extracts. Unfortunately, due to the presence of ethanol in *Centella asiatica* extract and the consequent pressure effect and explosion, we did not use *Centella asiatica* extract for high-performance microwave treatment. Therefore, the high-performance microwave treatment was used only for *Tilia tomentosa* extract, which we could not decontaminate against *B*. *zhangzhouensis* M1 with the harsh thermal treatment by the thermocycler. The results from the high-performance microwave technique revealed that the increase of temperature to 105°C for only 1 min could decontaminate the *Tilia tomentosa* extract (Table 4). This could decrease the thermal treatment duration dramatically, which directly affects the product quality. The mechanism behind the sporicidal activity of microwave technique (thermal or non-thermal) has been controversial among scientists [33]. The thermal effect is mainly related to the encounter of the dipole molecules such as water and cells by an electromagnetic field and the consequent friction and heating [17, 34]. Conversely, a non-thermal effect is solely linked to electromagnetic energy and not the heat from collision and friction [35]. Our study does not conclude that microwave heating could be more effective than the thermal cyclerone; however, the quick warm-up to the target temperature (105°C) and the consequent decontamination of the *Tilia tomentosa* extract was a critical achievement reached by the high-performance microwave technique.

**Table 4:** Effect of microwave treatments on *Bacillus* endospores inoculated in *Tilia tomentosa* extract.

Treatments	mixture of endospores
Initial number	4.26±0.01*
95°C-1min	2.99±0.01*
95°C-5min	1.98±0.03*
95°C-10min	1.68±0.25*
100°C-1min	2.93±0.06*
100°C-5min	1.10±0.17*
100°C-10min	<1.00**
105°C-1min	<1.00**
105°C-5min	<1.00**
105°C-10min	<1.00**

* Colonies were counted as CFU/ml and results are expressed as the mean (log_10_) ± SD (n = 3) of surviving endospores (Interaction: 0.0001,****/Time:0.0001,****/

Temperature:0.0001,****).

**The plates without colony were rechecked as CFU/mL to detect any surviving endospores, and the values remained the same (<1.00).

In this study, spores of *B*. *pumilus* T2 had higher thermal tolerance in *Tilia tomentosa* and *Centella asiatica* extracts compared to spores of *B*. *subtilis* and *B*. *zhangzhouensis*. In addition to the evaluation of single species, a mixture of endospores from the three strains, mimicking a possible natural contamination, was tested both with the thermocycler and the high-performance microwave treatments. The results from the microwave technique show that with the microwave technique it is possible to completely decontaminate the products using a much shorter treatment time, thanks to the speed with which the treatment temperature is reached. The results provided in this research considerably contribute to optimizing an original decontamination method for similar products containing glycerol and ethanol with the most negligible effect on product quality, which could be critical for many industries.

## Supporting information

S1 Data (XLSX)
